# Challenges and management of laparoscopic treatment of pyonephrosis caused by calculi

**DOI:** 10.1186/s12893-020-00992-5

**Published:** 2020-12-10

**Authors:** Jun Liu, Liang Chen, Lizhe An, Kai Ma, Xiongjun Ye, Qingquan Xu, Xiaobo Huang, Liulin Xiong

**Affiliations:** 1 Urology and Lithotripsy Center, Peking University People’s Hospital, 133 Fuchengmen inner Street, Beijing, 100034 People’s Republic of China; 2 Peking University Applied Lithotripsy Institute, Peking University, Beijing, 100034 People’s Republic of China

**Keywords:** Laparoscopy, Pyonephrosis, Infection, Renal stone

## Abstract

**Background:**

Calculous pyonephrosis is a disease characterized by infectious hydronephrosis associated with pyogenic destruction of the renal parenchyma, with complete or almost complete loss of renal function.

**Methods:**

The clinical data of laparoscopic nephrolithotomy performed at Peking University People’s Hospital from May 2017 to June 2020 were analyzed retrospectively. Eight patients (2 men; 6 women) aged 27 to 65 years (average age, 45.8 years) were included. Among them, 7 patients were treated with retroperitoneal approach and 1 patient by transperitoneal approach. All patients had received more than one endoscopic lithotripsy before nephrectomy. Renal dynamic imaging and computed tomography revealed the absence of function in pyonephrosis before nephrectomy. General clinical data and perioperative data were recorded. All nephrectomies were performed by the same physician.

**Results:**

Laparoscopic surgery was successfully performed in 7 patients; however, 1 patient underwent open surgery because of bleeding. The operation time, average operation time, and blood loss were 1.5–4.5 h, 3.4 h, and 100–1000 ml (average, 300 ml), respectively. The postoperative pathology showed inflammatory renal disease in 6 patients, xanthogranulomatous pyelonephritis in 1 patient, and high-grade urothelial cancer in 1 patient. The average postoperative hospital stay was 5.3 days. One patient had a Clavien–Dindo Grade IIIb complication (severe hematuria), which required laparotomy, and was found that there was bleeding of ureteral stump. None of the patients experienced poor healing of endoscopic wounds.

**Conclusion:**

For patients with complicated calculous pyonephrosis, renal inflammation could not be effectively controlled, and renal function was seriously damaged. Thus, kidneys should be immediately resected. With laparoscopy, patients may recover quickly, but surgeons require enough experience when performing laparoscopy to achieve safety.

## Background

Pyonephrosis is a disease characterized by infectious hydronephrosis associated with pyogenic destruction of the renal parenchyma, with complete or almost complete loss of renal function [1]. In recent years, with the widespread use of minimally invasive treatment for renal calculi, a large number of patients with renal calculi have been treated with percutaneous nephrolithotomy (PCNL) or ureteroscopic laser lithotripsy. For some calculi with pyogenic obstructive pyelonephritis, reasonable operation can usually achieve better results. However, when an irreversible renal function damage is observed, resection of the affected kidney is often required to avoid the inflammatory damage to the whole body. Moreover, the indications and timing of affected kidney resection deserve more extensive researches. Additionally, more attention is needed to answer the question of how to apply the laparoscopic minimally invasive technology to complex calculous pyonephrosis resection.

In the past 3 years, our center has treated 8 patients with complicated pyonephrosis caused by lithotripsy. The report is as follows.

## Methods

### Patients’ data

**Fig. 1:**
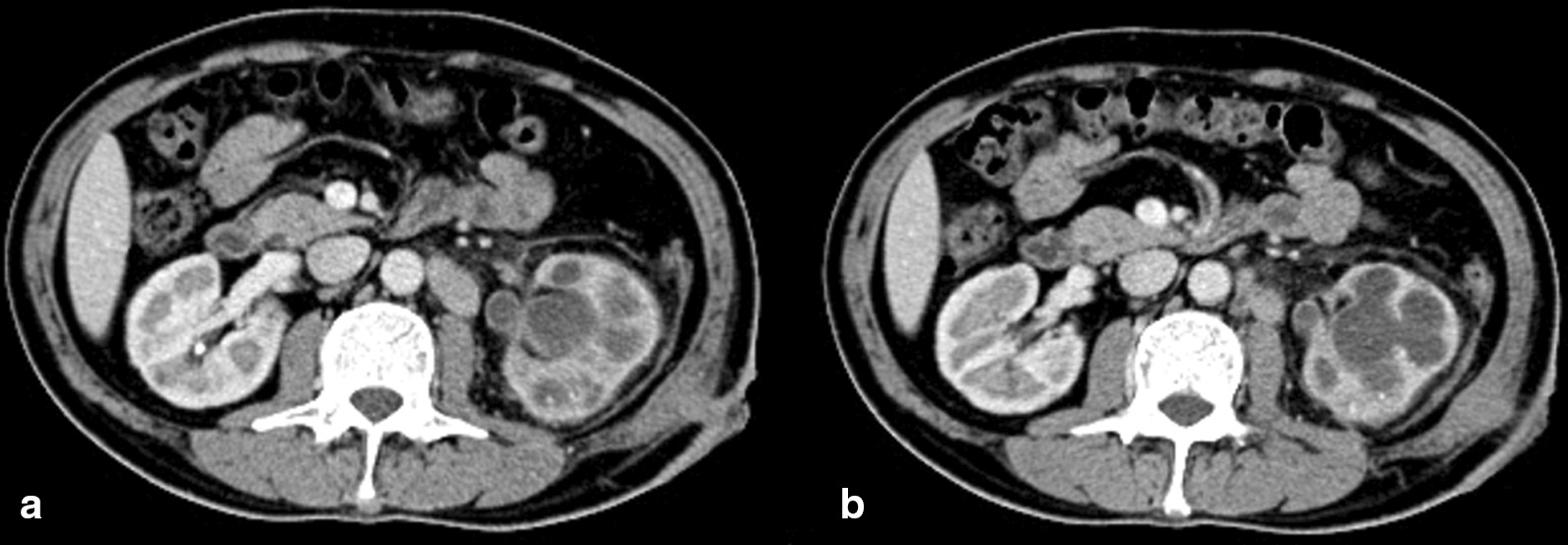
**a** Case 3 Computed tomography (CT) revealed atrophy of the left renal cortex, stone obstruction, and formation of the left perirenal sinus; **b** Enhanced CT revealed “bear’s paw” sign in the left kidney

From May 2017 to June 2020, we retrospectively analyzed 8 patients with pyonephrosis undergoing laparoscopic nephrectomy in our center. All nephrectomies were performed by the same physician with enough experience in endoscopic and laparoscopic treatment. The patients had good compliance and regular outpatient follow-up. Patients with the following characteristics were included in the study: (A) patients with history of PCNL or flexible ureteroscopy lithotripsy (FURL) (≥ 1 time); (B) patients with seriously damaged renal function defined as functional phase curve of renogram showing a low-level elongated line showed at preoperative renal dynamic examination; and (C) patients with renal cortex was evidently thinner or the renal parenchyma was severely damaged and the outflow tract of the renal pelvis and calyces was closed or narrow, with or without a long segment of ureter inflammatory thickening and with or without fistula formation showed at preoperative computed tomography (Fig. 1); (D) Internal or external drainage was performed for more than 6 weeks before nephrectomy. Eight (2, men; 6, women) patients aged 27 to 65 years (average age, 45.8 years) were included in this study. See Table 1 for detailed clinical data.

**Table 1:** Clinical data of 8 patients

No	Sex	Age (years)		Cr before Np, μmol/L	Infected side	Stone size (cm)	Grade of HDN	GFR before Np (left/right, ml/min)	PCNL or FURL before Np	Fistula	Urine culture
1	Male	55		104	Left	4.9	3	23.9/56.0	3 PCNL	Yes	Sterile
2	Female	27		77	Left	3.5	3	24.3/57.1	4 PCNL	No	*E. coli*
3	Female	32		74	Left	6.2	3	35.9/55.6	3 PCNL	Yes	*E. coli*
4	Female	65		66	Right	3.3	3	76.9/20.3	2 FURL	No	*E. coli*
5	Male	37		66	Right	2.6	2	47.3/19.4	2 FURL	Yes	Proteus
6	Female	41		103	Right	6.0	3	43.7/23.3	3 PCNL	No	Candida
7	Female	49		61	Left	6.8	2	11.2/51.6	1 PCNL	Yes	*E. coli*
8	Female	60		82	Right	8.5	3	70.2/16.1	1 PCNL	No	Proteus

Ultrasonographic grading of hydronephrosis (HDN) was defined as follows [2]: Grade 1 = subtle distension of the fornices to frank distension of the calices, with easily identifiable papillae; grade 2 = calices ballooned outward and papillae barely visible; grade 3 = marked HDN with a saclike collecting system and markedly thin parenchyma.

*Cr* creatinine, *Np* nephrectomy

### Operation method

Seven patients assumed the lateral decubitus position. First, a 2-cm-long longitudinal incision was made 1 cm above the iliac crest. Subsequently, the incision was expanded using the big bending forceps, the lumbodorsal fascia was bluntly punctured, and a self-made air bag was placed. The abdominal cavity space expanded after a 400–500 ml of air was injected. A 12-mm puncture trocar was inserted into the tip of the 11 ribs guided by the fingers, and a 5-mm puncture trocar was placed approximately 7 cm below it. After pneumoperitoneum was observed, a pressure of 13 mmHg and a carbon dioxide flow rate of 20 L/min were administered, and a 12-mm puncture trocar was placed through the costal ridge angle under direct vision. Edema of the retroperitoneal tissue was evident. Opening Gerota fascia in front of the psoas muscle, a heavy adhesion around the renal hilum was observed. The Hem-o-lok (Weck; Telefex Medical, Durham, NC, USA) horizontal clamp of the renal artery was used, subsequently closed, and severed. The ventral side of the kidney was separated along the renal capsule. If the adhesion was serious, it was dissociated under the renal capsule, and the ureter was separated at the level of the lower pole of the kidney. The connective tissue around the kidney was free from the renal fat capsule and free from the renal vein from the lower pole or ventral side of the kidney. Ligation and disconnection were performed using the same method. The kidney and perirenal fat were resected completely, and the adrenal gland was preserved. After the specimen was bagged, the two ventral incisions were completely connected and cut, and the specimen was completely removed. After active bleeding was not evidently observed, the retroperitoneal drainage tube was retained, and the incision was closed in layers.

One patient underwent transperitoneal laparoscopy, because there were 4 puncture scars in her right rank due to 3 PCNL this patient underwent before [3]. The biopsy pathology of the patient indicated urothelial carcinoma during the last PCNL procedure. However, because of the serious vascular adhesion during the treatment of the renal hilum, blood loss during the operation was up to 1000 mL. Hence, the patient underwent open surgery. Moreover, the right kidney and upper ureter were resected, and the middle and lower ureters were treated by a two-stage operation in plan. However, this patient developed severe hematuria on the 5th day after nephrectomy. After laparotomy, it was found that there was bleeding of ureteral stump. The patient was classified as IIIb according to Clavien-Dindo classification. After operation, the patient recovered well and was discharged from hospital 7 days after the second operation.

## Results

**Fig. 2:**
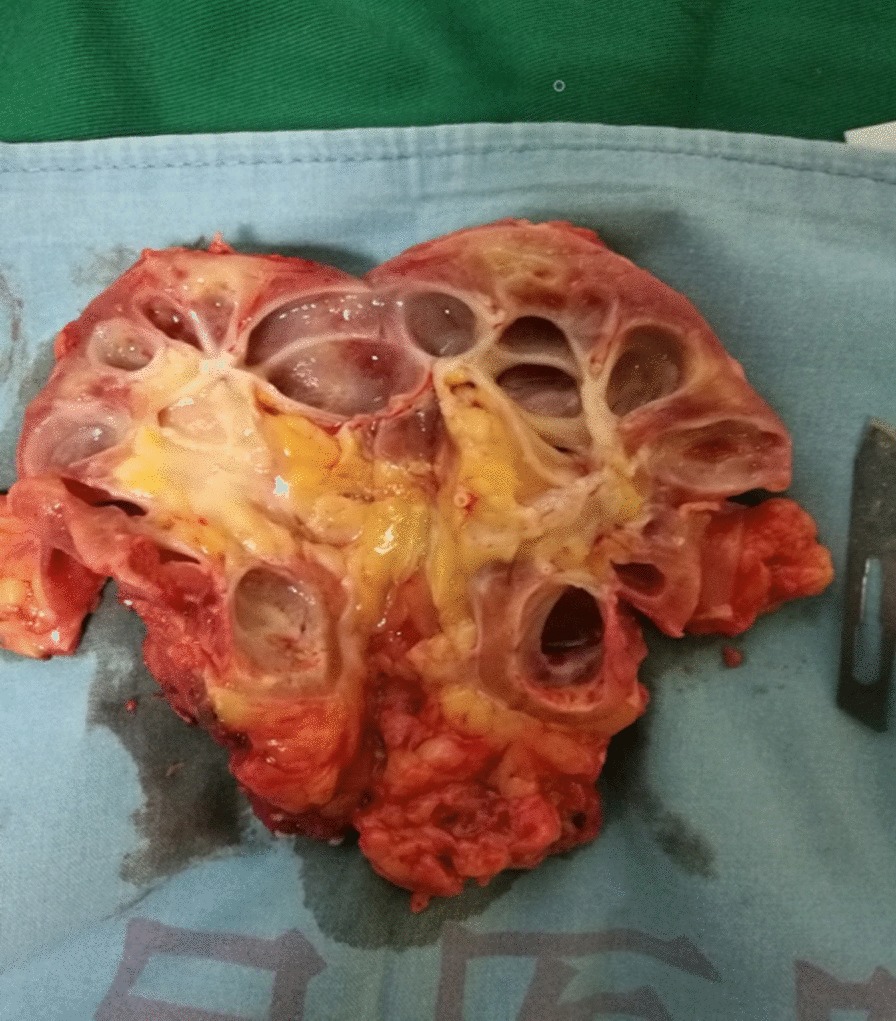
Case 3 Renal parenchymal fibrosis, deposition of necrotic substances in the renal calices, and poor drainage can be observed in the gross specimen of left nephrectomy

In this group, 7 patients were successfully treated with laparoscopic surgery, and 1 patient underwent open surgery because of bleeding. The operation time, average operation time, and blood loss were 1.5–4.5 h, 3.4 h, and 100–1000 ml (average, 300 ml), respectively. The postoperative pathology showed inflammatory renal disease in 6 patients. The gross specimen showed renal fibrosis, deposition of necrotic material in the renal calices, and poor drainage (Fig. 2). Moreover, one patient had xanthogranulomatous pyelonephritis, and another patient had high-grade urothelial carcinoma. The average hospital stay was 5.3 days, the average follow-up was 26 months. One patient had a Clavien–Dindo Grade IIIb complication (severe hematuria), which required laparotomy, and was found that there was bleeding of ureteral stump. None of the patients experienced poor healing of endoscopic wounds. (Table 2).

**Table 2:** Operation information of patients

No	Operation method	Operation time, hours	Blood loss, ml	Creatinine after nephrectomy, μmol/L	Pathology	Hospital days after nephrectomy
1	Retrop	2	100	87	Atrophic inflammatory kidney	3
2	Retrop	3.5	200	80	Atrophic inflammatory kidney	5
3	Retrop	4	300	69	Atrophic inflammatory kidney	3
4	Retrop	4.5	200	57	Atrophic inflammatory kidney	4
5	Retrop	2.5	100	71	Xanthogranulomatous pyelonephritis	5
6	Transp	3.5	1000	117	High-grade urothelial carcinoma	5
7	Retrop	3.5	50	75	Atrophic inflammatory kidney	5
8	Retrop	4	100	89	Atrophic inflammatory kidney	12

*Retrop* retroperitoneal laparoscopy, *Transp* transperitoneal laparoscopy

## Discussion

Considering the diversification and popularization of minimally invasive treatment methods for renal calculi, most renal calculi can achieve good diagnosis and treatment. According to the European Association of Urology guidelines, the main treatments for renal calculi measuring < 2 cm should include extracorporeal shock wave lithotripsy (ESWL) and FURL. The main treatment for renal calculi measuring > 2 cm should include percutaneous nephrolithotomy (PCNL) [4]. If adverse effects are observed in ESWL, FURL or PCNL should be performed in patients with 1–2-cm lower pole stones. At the same time, we also note that not all patients with renal calculi can achieve good recovery of renal function after the removal of obstruction. Sometimes, in some patients, renal functions consistently deteriorate after surgery, but these cases are rarely reported in the literature.

Pyonephrosis is a disease characterized by the pyogenic destruction of the renal parenchyma caused by the progression of infectious hydronephrosis. In pyonephrosis, renal function is almost completely lost. Infectious hydronephrosis refers to hydronephrosis with bacterial infection. Notably, the end stage of infectious hydronephrosis can be considered as the initial stage of pyonephrosis [1]. However, there is no clear predictive model demonstrating the occurrence of pyonephrosis in patients with kidney stones. Patodia retrospectively analyzed 501 patients with kidney stones and found that some factors were closely associated with the occurrence of pyonephrosis, including larger stone volume, severe hydronephrosis, poor renal function, past history of urological surgery, and obstruction caused by other causes than stones [5]. These are considered beneficial in determining which patients with kidney stones are prone to pyonephrosis. In our study, all patients underwent more than one lithotripsy, all of whom had moderate to severe hydronephrosis, including one patient with ureteral junction stenosis due to long-term inflammatory stimulation. Therefore, surgery and follow-up should be performed from the initial stage of stone treatment in patients with these risk factors. The patients included in this study were all diagnosed with pyonephrosis caused by stones. These patients experienced irreversible kidney damage due to long-term concurrent infection with stones. After several operations, the patients’ kidney functions were damaged, and the whole kidney even experienced infection, which continuously produced a chronic inflammatory response to the body. Some patients are prone to urinary tract tumors with long-term chronic stimulation of inflammation [6]. Current studies suggest that patients with long-term kidney stones are closely associated with the occurrence of urothelial carcinoma [7–9]. Therefore, for patients with complex renal calculi and long course of disease, it is necessary to pay attention to whether there is renal pelvis tumor in the process of diagnosis and treatment. We believe that for patients with long-term renal stone infection, we should pay attention to nonspecific symptoms such as low back pain, fever and anemia before operation, and should not simply be attributed to urinary tract infection and ignore the possibility of tumor. If the renal function is normal, enhanced CT scanning of urinary system is helpful to detect tumor. Intraoperative soft tissue biopsy is necessary for suspicious mucosal lesions. In this study, a patient with pyonephrosis complicated with renal pelvic tumors was found to have suspicious lesions in the renal pelvis during first-stage PCNL operation, which is difficult to distinguish from pyonephrosis. The possible difference is that the neoplasm can bleed when the lesion is touched, while a lesion in pyonephrosis does not easily bleed. This patient was diagnosed by pathological biopsy, and laparoscopic nephrectomy was performed in a two-stage operation. Therefore, for patients with calculous pyonephrosis, specifically those with a long history, attention should be paid to the presence of combined renal pelvic tumors.

Whether kidney with pyonephrosis should be preserved is directly related to actual renal function. Regarding the judgment of renal function, the majority of literatures take renal dynamic imaging as the basis of evaluation, and the glomerular filtration rate (GFR) is considered to be nonfunctional when it is less than 10–15 ml/min [10–12]. However, in our study, we found that 8 patients had pyonephrosis, and their GFR was between 11.2 and 35.9 ml/min. Based on the postoperative pathological analysis, calculous pyonephrosis usually has a large number of lymphocyte infiltration, severe local inflammation, a large number of renal parenchymal fibrosis, and glomerular atrophy and does not have normal filtration function. Therefore, the value of GFR alone does not reflect the real renal function of patients. Further, it cannot be used as the sole basis for kidney preservation or nephrectomy. At present, renal dynamic imaging is the most widely used method for evaluating renal function. Often, we directly focused on the final GFR values of the patients’ both kidneys, which indeed directly reflects the true renal function in nonobstructive diseases. However, when applied to the judgment of renal function in stone obstruction, renal function is usually severely impaired, and renal dynamic imaging can easily lead to the overestimation of the actual glomerular filtration function of the affected kidney [13]. Currently, we need to pay attention to the curve shape of the GFR. In general, renal dynamic imaging is divided into the perfusion phase and functional phase. When the renal blood flow function phase is not clearly developed, it is recognized as a low-level prolongation line. In fact, the kidney has no filtering function. In combination with the imaging characteristics of enhanced CT, the renal pelvis of the affected kidney is reduced, the calyx is dilated, and the cortex is thinned and resembles a bear’s footpad (Fig. 1). Hence, it is called the “bear’s paw sign” [14]. Subramanyam summarized the ultrasonographic features of 73 patients with hydronephrosis, in which the ultrasonographic diagnosis of pyonephrosis was characterized by persistent low-to-moderate internal echoes within dilated collecting system, and concluded that ultrasonography had a sensitivity of 90% and a specificity of 97% for the diagnosis of pyonephrosis. This plays an important role in determining the occurrence of pyonephrosis based on several aspects [15].

Concerning surgical resection indication for calculous obstructive pyonephrosis, besides the above evaluation of renal function, we considered that the following factors should be considered equally: (A) recurrent stones and poor control of infection; (B) multiple calyx neck atresia or stenosis in the kidney, which cannot be effectively treated by endoscopy; and (C) thickening of the long ureteral wall or iatrogenic long-segment ureteral stenosis. Therefore, when the treatment results of pyonephrosis due to calculi is not satisfactory and the infection control of the affected kidney is insufficient, PCNL or FURL should not be performed again. Focusing solely on the GFR value of the affected kidney to judge renal function should be avoided. A correct surgical plan should be taken into consideration in combination with the dynamic phase curve of kidney function, kidney stones, infection, and contralateral kidney function. Retaining the affected kidney blindly may cause the patient’s condition to be delayed, increase the cost of medical treatment, and negatively affect the patient’s quality of life.

After multiple PCNL or FURL it is important to determine the correct timing of laparoscopic treatment. In the stage of acute infection, internal or external drainage should be performed first, and surgical treatment should be started at least 6 weeks after adequate drainage. In the anti-infection treatment stage, attention should be paid to the patient’s renal function and drainage of the affected kidney. All patients completed the urine culture before laparoscopic nephrectomy, for the patients with positive urine culture before operation, we gave adequate course of antibiotic treatment. After two times of negative urine culture and no fever, the operation was carried out. For patients without systemic inflammation before operation, only prophylactic antibiotic treatment was given before operation. There was no postoperative fever in this group. However, once postoperative fever occurred, the results of urine culture can help us guide the treatment of patients with postoperative infection. For urologists, nonfunctional pyonephrectomy after endoscopic interventions remains a challenging procedure, with Duarte reporting a 72% success rate for laparoscopic resection of nonfunctional pyonephrosis alone [16].

Although transperitoneal approach is easy to establish, has a large operative space and evident anatomical markers, and is conveniently performed when dealing with intraoperative complications, this approach may be more difficult in dealing with renal hilar vessels for pyonephrosis after endoscopic interventions because there could be severe adhesion between the kidney and psoas major muscle. One patient in this group underwent a transperitoneal approach, but when dealing with renal hilar vessels, the patient was forced to undergo open surgery because of excessive bleeding. The retroperitoneal laparoscopic approach is mostly used in our center. We believe that the retroperitoneal approach has significant advantages including the following. First, with the retroperitoneal laparoscopic treatment of pyonephrosis, the abdominal cavity cannot be accessed; hence, intraoperative renal rupture can be avoided and the risk of abdominal infection is reduced. Second, when isolating the renal artery, the retroperitoneal approach is simpler than the abdominal approach due to its anatomical relationship with the renal artery. Third, in our review of 8 patients, 4 were treated after PCNL with fistula tract purulence, and with the retroperitoneal approach, it can debride the fistula tract immediately after the resection of the kidney and reduce the patient’s trauma.

It should be noted that the establishment of the retroperitoneal space is more difficult compared to the conventional retroperitoneal laparoscopic surgery because repeated endoscopy and inflammatory stimulation of pyonephrosis and inflammatory adhesion of the retroperitoneal space are often severe, and the establishment of the retroperitoneal space can easily cause unclear layers and wound bleeding. In fact, the establishment of pneumoperitoneum in the conventional retroperitoneal space, we usually establish the first puncture passage by puncture expansion above the iliac crest, where there is usually a thick extraperitoneal fat as a liner, and where the inflammatory adhesion is mild. Because of the long-term and repeated inflammatory stimulation around the renal artery, it is difficult to detect the renal artery directly from the separated renal hilum, and the surgical field is unclear. Our study aimed to detect the psoas major muscle plane first, along with the psoas major muscle plane, from the lower pole of the kidney to the renal hilum for freeing, to clearly dissect the renal artery after the renal artery is disconnected. In the process of handling the renal vein, the position of the renal portal is relatively fixed, and the space is insufficient. At this time, the upper and lower poles of the kidney need to be dissected freely. After the whole kidney has a certain range of activity, the renal vein should be clearly exposed and subsequently severed. If the adhesion between the ventral and upper poles of the patient is serious, subcapsular resection can be performed.

Considering the data from Table 3, the mean operation time of pyonephrosis of our center is generally longer than that of previous literatures (3.4 h vs. 2.0 h) [3, 10, 11, 17–19], which is closely associated with previous PCNL or FURL, adhesions of renal hilum structure, and unclear layers caused by multiple surgical disturbances. Therefore, surgeons should have enough experience in performing retroperitoneal laparoscopic surgery.

**Table 3:** Comparison of perioperative results using laparoscopic nephrectomy for inflammatory kidney with renal stone

References	N	Previous surgical history	Preoperative GRF	Method	Conversion	Mean operative time(min)	Hospital days after nephrectomy
Hemal, et al. [10]	46	PCN	< 10 ml/min	Retrop	6	110	3.6
Tepeler, et al. [17]	27	None	–	Retrop	2	123	3.1
Kaba, et al. [3]	15	None	–	Transp	1	95	2.9
Kurt, et al. [18]	22	None	< 10%, at DSMA renal scan	Transp	0	130	3.1
Naghiyev, et al. [19]	63	5 PCNL, 2 URL	< 10%, at DSMA renal scan	44 Transp, 19 Retrop	3	109	4.1
Wang, et al. [11]	33	PCN	< 15 ml/min	Retrop	4	162	3.1

*Retrop* retroperitoneal laparoscopy, *Transp* transperitoneal laparoscopy, *PCN* percutaneous nephrostomy, *URL* ureteroscopic lithotripsy, *DSMA* Dimercaptosuccinic acid

## Conclusion

In summary, for patients with complex kidney stones, once the stones lead to the outcome of nonfunctional pyonephrosis, blindly retaining the kidney may worsen the disease. Timely nephrectomy can effectively control inflammation, and with retroperitoneal laparoscopy, patients may recover quickly. However, retroperitoneal laparoscopic nephrectomy for pyonephrosis has a certain complexity, and surgeons should have sufficient and extensive experiences when performing laparoscopy to achieve safety.

## Data Availability

The datasets used and analyzed during the current study are available from the corresponding author on reasonable request.

## Abbreviations

PCNL: Percutaneous nephrolithotomy
FURL: Flexible ureteroscopy lithotripsy
Retrop: Retroperitoneal laparoscopy
Transp: Transperitoneal laparoscopy
ESWL: Extracorporeal shock wave lithotripsy
GFR: Glomerular filtration rate
CT: Computed Tomography
